# Comparative toxicity and survival dynamics of paraquat and a glyphosate-based formulation in adult *Drosophila melanogaster*

**DOI:** 10.1016/j.toxrep.2026.102365

**Published:** 2026-10-29

**Authors:** Raphael Mwezi, Flora Stephano, Maulilio Kipanyula, John-Mary Vianney

**Affiliations:** a Directorate of Pesticides, Tanzania Plant Health and Pesticide Authority (TPHPA), Arusha, Tanzania; b School of Life Sciences and Bioengineering (LiSBE), Nelson Mandela African Institution of Science and Technology, Arusha, Tanzania; c Department of Zoology and Wildlife Conservation College of Natural and Applied Sciences, University of Dar es Salaam (UDSM), Dar es Salaam, Tanzania

**Keywords:** Paraquat, Roundup 360 SL, Glyphosate acid equivalent, *Drosophila melanogaster*, Mortality, Survival analysis, Concentration–mortality relationship

## Abstract

Paraquat and glyphosate-based herbicides are widely used, but their toxic effects depend on concentration, formulation, and exposure conditions. We evaluated seven-day mortality and survival in adult male *Drosophila melanogaster* exposed through treated food to TwigaQuat® 200 SL or Roundup™ 360 SL. Each experiment comprised three independent biological phases, with three replicate vials per treatment per phase and 20 flies per vial (nine vials and 180 flies per treatment). Paraquat-equivalent target concentrations were 0, 10, 20, 30, and 40 mM. Roundup 360 SL was tested at target concentrations of 0, 0.5, 1.0, and 2.0 g/L glyphosate acid equivalent (GAE). Vial-level endpoint mortality was analysed using phase-adjusted beta-binomial and binomial models, with Dunnett-adjusted comparisons; temporal effects were evaluated using discrete-time hazard and phase-stratified Cox models. Paraquat mortality increased from 17.2% in controls to 28.3%, 37.2%, 51.7%, and 67.2% at 10–40 mM, respectively. The estimated LC50 was 28.1 mM (95% CI 24.7–32.6), and mortality hazard increased progressively with concentration. For Roundup 360 SL, mortality was 13.3%, 22.2%, 27.8%, and 26.7% at 0, 0.5, 1.0, and 2.0 g/L GAE, respectively; endpoint mortality was significantly higher at 1.0 and 2.0 g/L GAE, while the two highest treatments produced similar mortality and hazards. This upper-range flattening does not establish hormesis or a reproducible non-monotonic response. Because Roundup was tested as a complete commercial formulation, its effects cannot be attributed specifically to glyphosate. Direct relative-potency comparison between the two herbicides was not attempted because exposure metrics and formulation composition differed.

## Highlights

• Paraquat caused a clear concentration-related increase in mortality and hazard.
• Seven-day mortality reached 67.2% at 40 mM paraquat.
• Glyphosate-based formulation mortality plateaued at higher exposure levels.
• Formulation effects cannot be attributed specifically to glyphosate alone.
• Vial-level replication and survival analysis strengthened toxicity assessment.

## Graphical Abstract

**Figure:**
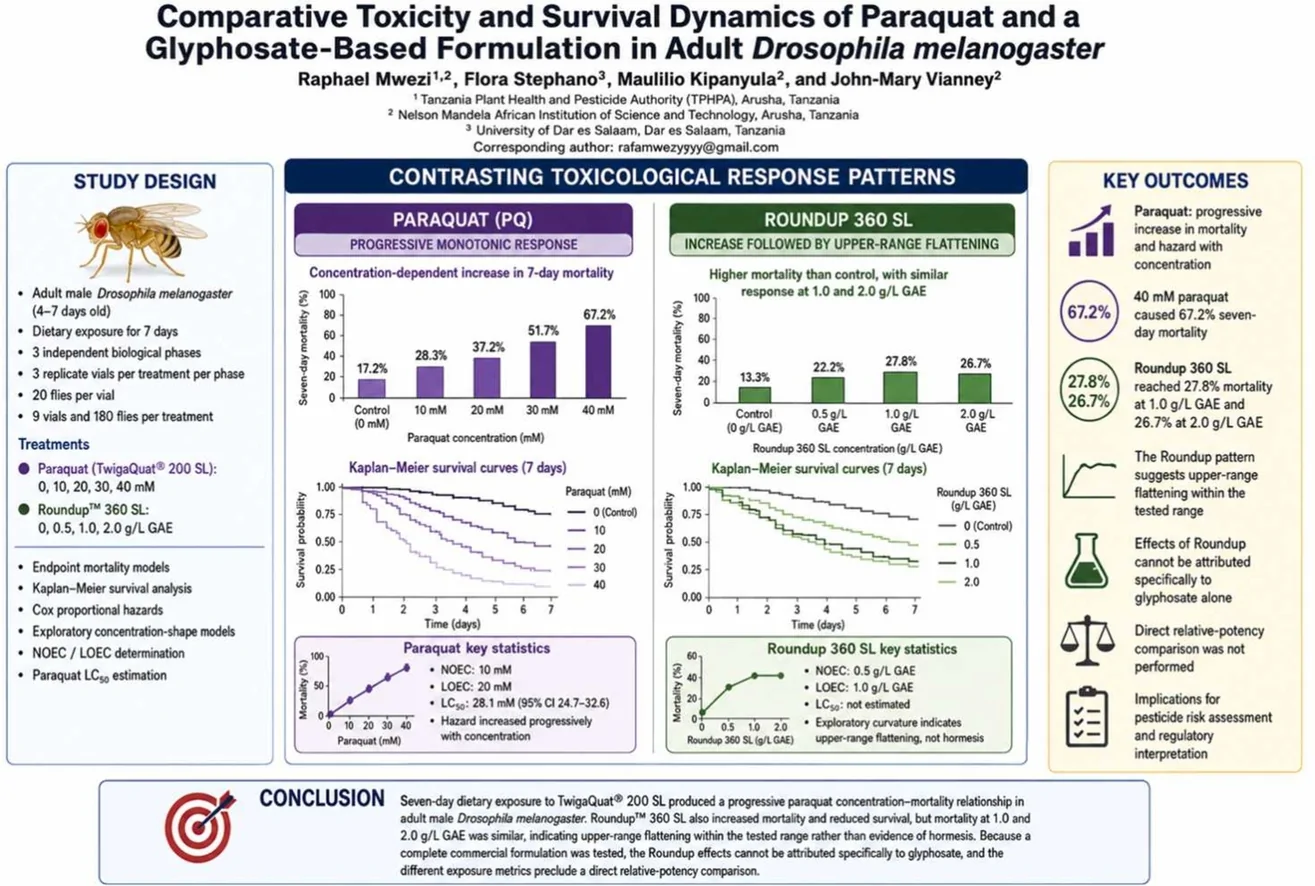

## 1 Introduction

Herbicides are central to modern weed management, and the large scale of glyphosate use has intensified interest in possible effects on non-target organisms [1]. Paraquat and glyphosate-based herbicides differ substantially in chemistry and toxicological action, Paraquat undergoes redox cycling and can generate intracellular oxidative stress, a mechanism implicated in its toxicity [2], [3], [4]. In *Drosophila melanogaster*, paraquat exposure has been used to model oxidative and neurotoxic stress and has been used to investigate survival and functional outcomes [5], [6], [7], [8]. Commercial glyphosate-based herbicides require formulation-level interpretation because glyphosate salts are combined with co-formulants that can modify uptake and toxicity [9], [10], [11]. Experimental effects observed after exposure to a commercial product therefore cannot be assigned to glyphosate alone unless pure glyphosate and suitable formulation or co-formulant controls are included. Drosophila studies have reported adverse effects of Roundup formulations on survival or reproduction, further supporting evaluation of the complete formulation rather than the active ingredient in isolation [9], [12].

*Drosophila melanogaster* is a useful toxicological model because of its short life cycle, genetic tractability, conserved cellular pathways, and suitability for repeated survival observations [13], [14]. In the present study, paraquat and Roundup 360 SL were evaluated in separate experiments under a common biological model. The objective was not to rank the two products by chemical potency, because their exposure concentrations were expressed on different bases and individual intake was not measured. Instead, we compared within-experiment patterns of endpoint mortality, survival probability, and mortality hazard. A plateau in mortality was treated as an exploratory response pattern and not as evidence of hormesis unless a reproducible biphasic response with a beneficial or stimulatory low-exposure component could be demonstrated.

Accordingly, this study aimed to (i) quantify seven-day mortality in adult male *D. melanogaster* exposed to paraquat or Roundup 360 SL; (ii) evaluate treatment effects using replicate-vial data across three independent biological phases; (iii) characterize temporal mortality and survival dynamics; (iv) determine operational seven-day endpoint no-observed-effect and lowest-observed-effect concentrations (NOEC and LOEC); (v) estimate the paraquat LC50 where supported by the tested concentration range; and (vi) compare the shapes of the within-experiment toxicity patterns without inferring direct relative potency or hormesis.

## 2 Materials and methods

### 2.1 Study design and experimental structure

Two separate controlled laboratory experiments evaluated seven-day mortality and survival after dietary exposure to paraquat or Roundup 360 SL. Each experiment comprised three independent biological runs (phases). Within each phase, every treatment was represented by three replicate vials containing 20 flies each; consequently, each treatment comprised nine vials and 180 flies across the three phases. The paraquat experiment included five treatment groups (45 vials; 900 flies), and the Roundup experiment included four treatment groups (36 vials; 720 flies). The replicate vial was the experimental unit for endpoint analyses; individual flies sharing a vial were treated as subsamples. For time-to-event analyses, individual event times were used while within-vial dependence was accounted for by the survival models.

### 2.2 Experimental organism and maintenance

Wild-type Oregon K *D. melanogaster* were obtained from the Department of Zoology and Wildlife Conservation, University of Dar es Salaam, Tanzania. Flies were maintained at 22 ± 1 °C, 60–70% relative humidity, and a 12:12 h light: dark photoperiod. Only male flies aged 4–7 days were included, thereby controlling sex across all treatment groups. Before exposure, flies were randomly allocated to control and treatment vials [15].

### 2.3 Test substances and exposure-media preparation

#### 2.3.1 Paraquat formulation

Paraquat exposure was prepared using TwigaQuat® 200 SL, a commercial soluble-concentrate herbicide labelled to contain 200 g/L paraquat (registration no. RE/0148) and distributed in Tanzania by Twiga Chemical Industries (T) Ltd., Dar es Salaam. Because a commercial formulation rather than analytical grade paraquat was used, reagent purity and catalogue number were not applicable. For molar calculations, concentrations were expressed as paraquat equivalent using the molecular mass of 186.65 g/mol. Accordingly, the declared concentration of 200 g/L corresponded to an operational stock concentration of approximately 1.07 mol/L paraquat equivalent. Using the dilution relationship C₁V₁ = C₂V₂, approximately 168, 336, 505 and 673 µL of TwigaQuat 200 SL were used to prepare the nominal target concentrations of 10, 20, 30 and 40 mM paraquat equivalent, respectively. Each formulation aliquot was incorporated into culture medium, and the mixture was adjusted to a final total volume of 18.000 mL. Thus, 18.000 mL represents the final volume of the medium–formulation mixture, rather than the volume of medium before formulation addition. Untreated culture medium served as the concurrent control. Statistical analyses and treatment labels use the nominal target concentrations of 0, 10, 20, 30 and 40 mM paraquat equivalent. These concentrations were calculated from the declared formulation concentration and recorded preparation volumes; the exposure media were not chemically analysed.

#### 2.3.2 Glyphosate-based formulation

Exposure to the glyphosate-based formulation was prepared using Roundup™ 360 SL herbicide (registration no. HE/0055). The archived product information identifies Monsanto Kenya Limited as the registrant, Bayer Life Science Tanzania as the importer, and Bayer Agriculture BV, Haven 627, Belgium, as the manufacturer. The product label states 441 g/L potassium salt of glyphosate, equivalent to 360 g/L glyphosate acid. A detailed co-formulant composition was not recoverable from the records. No pure-glyphosate or co-formulant-matched control was included; therefore, all observed effects are attributed to the complete Roundup 360 SL formulation and not specifically to glyphosate. The laboratory preparation record showed that 25, 50, and 100 µL of Roundup 360 SL were added to 18.000 mL of culture medium. With the declared 360 g/L glyphosate acid-equivalent concentration, these aliquots contain 9, 18, and 36 mg GAE, respectively. The exact final mixture volumes were 18.025, 18.050, and 18.100 mL, giving constructed concentrations of 0.499, 0.997, and 1.989 g/L GAE, respectively. These values correspond to the intended treatment levels of 0.5, 1.0, and 2.0 g/L GAE used in the dilution formula rather than percentage concentrations. For completeness, the same mixtures contain approximately 0.612, 1.222, and 2.436 g/L glyphosate potassium salt and approximately 0.139%, 0.277%, and 0.552% (v/v) commercial formulation. Statistical analyses use the target GAE concentrations 0, 0.5, 1.0, and 2.0 g/L; the constructed exact values document the final exposure volumes. All Roundup treatment concentrations in this article, including tables and figures, are expressed as g/L glyphosate acid equivalent (GAE).

### 2.4 Exposure protocol

Adult male *D. melanogaster* aged 4–7 days were randomly allocated to treatment and concurrent-control vials and exposed continuously through treated food for seven consecutive days [6], [13]. In each phase, three replicate vials containing 20 flies each were used per treatment. Paraquat treatments were 0, 10, 20, 30, and 40 mM paraquat equivalent [5]. Roundup 360 SL treatments were 0, 0.5, 1.0, and 2.0 g/L GAE [16]. Control vials received the same culture medium without herbicide. All vials were maintained at 22 ± 1 °C, 60–70% relative humidity, and a 12:12 h light: dark photoperiod [17], [18]. The three phases were treated as independent biological runs in experimental design and statistical analysis. Sex was controlled by including only males in all groups.

### 2.5 Mortality and survival assessment

Mortality was assessed every 24 h for seven consecutive days. A fly was classified as dead when it showed no movement and no response to gentle mechanical stimulation. Dead flies were counted and removed after each observation. Vial-level seven-day mortality and survival were calculated from the number of flies initially introduced into each vial.

$$\mathrm{Seven}-\mathrm{day}\;\mathrm{mortality}(\%)=\frac{\text{Number}\;\text{of}\;\text{deaths}\;\text{by}\;\text{Day}\;7}{\text{Initial}\;\text{number}\;\text{of}\;\text{flies}\;\text{in}\;\text{the}\;\text{vial}}\times 100$$

Survival percentage was calculated as:

$$\mathrm{Seven}-\mathrm{day}\;\mathrm{survival}(\%)\;=\frac{\text{Number}\;\text{of}\;\text{flies}\;\text{alive}\;\text{on}\;\text{Day}\;7}{\text{Initial}\;\text{number}\;\text{of}\;\text{flies}\;\text{in}\;\text{the}\;\text{vial}}\times 100$$

### 2.6 Statistical analysis

All analyses were conducted separately for the paraquat and Roundup 360 SL experiments using R version 4.5.3. Seven-day endpoint mortality was analysed at the replicate-vial level, and experimental phase was included to account for the three independent biological runs. Statistical tests were two-sided with α = 0.05; effect estimates are reported with 95% confidence intervals.

#### 2.6.1 Seven-day endpoint mortality

Endpoint mortality was analysed from the numbers of dead and surviving flies in each replicate vial. Treatment was the principal explanatory variable, and experimental phase was included as a blocking factor. Paraquat data showed overdispersion and were analysed using a logit-link beta-binomial model; Roundup 360 SL data were analysed using a phase-adjusted binomial generalized linear model because important residual overdispersion was not detected. Treatment effects were expressed as odds ratios (ORs) with 95% confidence intervals. ANOVA and Kruskal–Wallis tests were not retained as primary inferential analyses because they do not directly model the binomial, clustered structure of vial-level mortality. Each non-zero treatment was compared with its concurrent control using Dunnett-adjusted contrasts. For the seven-day endpoint, the operational seven-day endpoint NOEC was defined as the highest tested non-zero concentration not significantly different from control after multiplicity adjustment, and the operational seven-day endpoint LOEC as the lowest tested concentration significantly different from control. These designations apply only to the predefined seven-day endpoint mortality analysis and do not imply absence of effects in time-to-event analyses.

#### 2.6.2 Concentration–mortality analysis

For paraquat, concentration was additionally modelled as a continuous predictor in a phase-adjusted beta-binomial model to quantify the concentration–mortality trend. The trend was summarized as the change in mortality odds per 10 mM increase. The LC50 was defined as the paraquat-equivalent concentration at which the fitted model predicted 50% seven-day mortality. Its 95% confidence interval was estimated from 5000 parametric bootstrap simulations. Because the estimated LC50 fell within the tested range, the estimate was treated as interpolation rather than unsupported extrapolation. For Roundup 360 SL, treatment was retained as a categorical factor in the primary endpoint analysis because only three non-zero GAE concentrations were tested. Linear and quadratic models using the target concentrations 0, 0.5, 1.0, and 2.0 g/L GAE were fitted only as exploratory analyses of response shape and compared using Akaike's information criterion (AIC) and likelihood-ratio testing. Curvature was not interpreted as proof of hormesis or a reproducible non-monotonic response. An LC50 was not estimated because mortality remained well below 50% over the tested range.

#### 2.6.3 Temporal mortality and survival analysis

Daily mortality was analysed using a complementary log–log discrete-time hazard model. Treatment, exposure day, and experimental phase were included as fixed effects, and replicate vial was included as a random intercept to account for repeated observations and clustering within vials. Treatment-by-day interactions were evaluated using likelihood-ratio tests to assess whether relative treatment effects changed over the seven-day exposure period. Kaplan–Meier curves were used to describe survival over time. Treatment-associated mortality hazards were estimated using Cox proportional-hazards regression stratified by experimental phase, with robust standard errors clustered by replicate vial. Global survival differences were assessed using the log-rank test, and the proportional-hazards assumption was evaluated using Schoenfeld residuals.

#### 2.6.4 Model diagnostics and reporting

Model adequacy was evaluated using residual uniformity, dispersion, and outlier diagnostics, together with inspection of the relevant model assumptions. Exact p-values are reported where appropriate, with p < 0.001 used for smaller values. Statistical evidence from exploratory curvature models was interpreted as evidence of model shape only and not as proof of hormesis.

## 3 Results

### 3.1 Paraquat toxicity

#### 3.1.1 Seven-day endpoint mortality

The paraquat experiment comprised 45 replicate vials and 900 flies, with nine vials and 180 flies per treatment. Seven-day mortality increased progressively from 17.2% in the control to 28.3%, 37.2%, 51.7%, and 67.2% at 10, 20, 30, and 40 mM, respectively (Table 1, Fig. 1). The phase-adjusted beta-binomial model showed a significant overall treatment effect (χ² = 40.11, df = 4, p < 0.001). Dunnett-adjusted comparisons showed no significant endpoint difference at 10 mM (OR = 1.82, 95% CI 0.81–4.10; adjusted p = 0.220), whereas mortality was significantly higher at 20 mM (OR = 2.64, 95% CI 1.18–5.89; p = 0.011), 30 mM (OR = 4.89, 95% CI 2.22–10.74; p < 0.001), and 40 mM (OR = 9.17, 95% CI 4.10–20.54; p < 0.001). The operational seven-day endpoint NOEC was therefore 10 mM and the LOEC was 20 mM. The treatment-by-phase interaction was not significant (χ² = 1.20, df = 8, p = 0.997), indicating no evidence that the relative concentration–mortality pattern differed among phases.

**Table 1:** Seven-day mortality of adult *Drosophila melanogaster* following paraquat exposure.

Paraquat concentration	Replicate vials (n)	Deaths/initial flies	Observed mortality (%)	Model-estimated mortality, % (95% CI)	Dunnett-adjusted OR (95% CI)	Adjusted *p*-value
Control (0 mM)	9	31/180	17.2	18.1 (11.9–26.5)	Reference	—
10 mM	9	51/180	28.3	28.6 (20.7–38.0)	1.82 (0.81–4.10)	0.220
20 mM	9	67/180	37.2	36.8 (27.9–46.7)	2.64 (1.18–5.89)	0.011
30 mM	9	93/180	51.7	51.9 (42.2–61.4)	4.89 (2.22–10.74)	< 0.001
40 mM	9	121/180	67.2	66.9 (57.3–75.3)	9.17 (4.10–20.54)	< 0.001

**Note:** OR, odds ratio; CI, confidence interval. Estimates were obtained from the phase-adjusted beta-binomial model. Adjusted p-values are from Dunnett comparisons of each concentration with the concurrent control. The replicate vial was the experimental unit. The underlying values in V4 are supported by the reanalysed vial-level dataset.

**Fig. 1:**
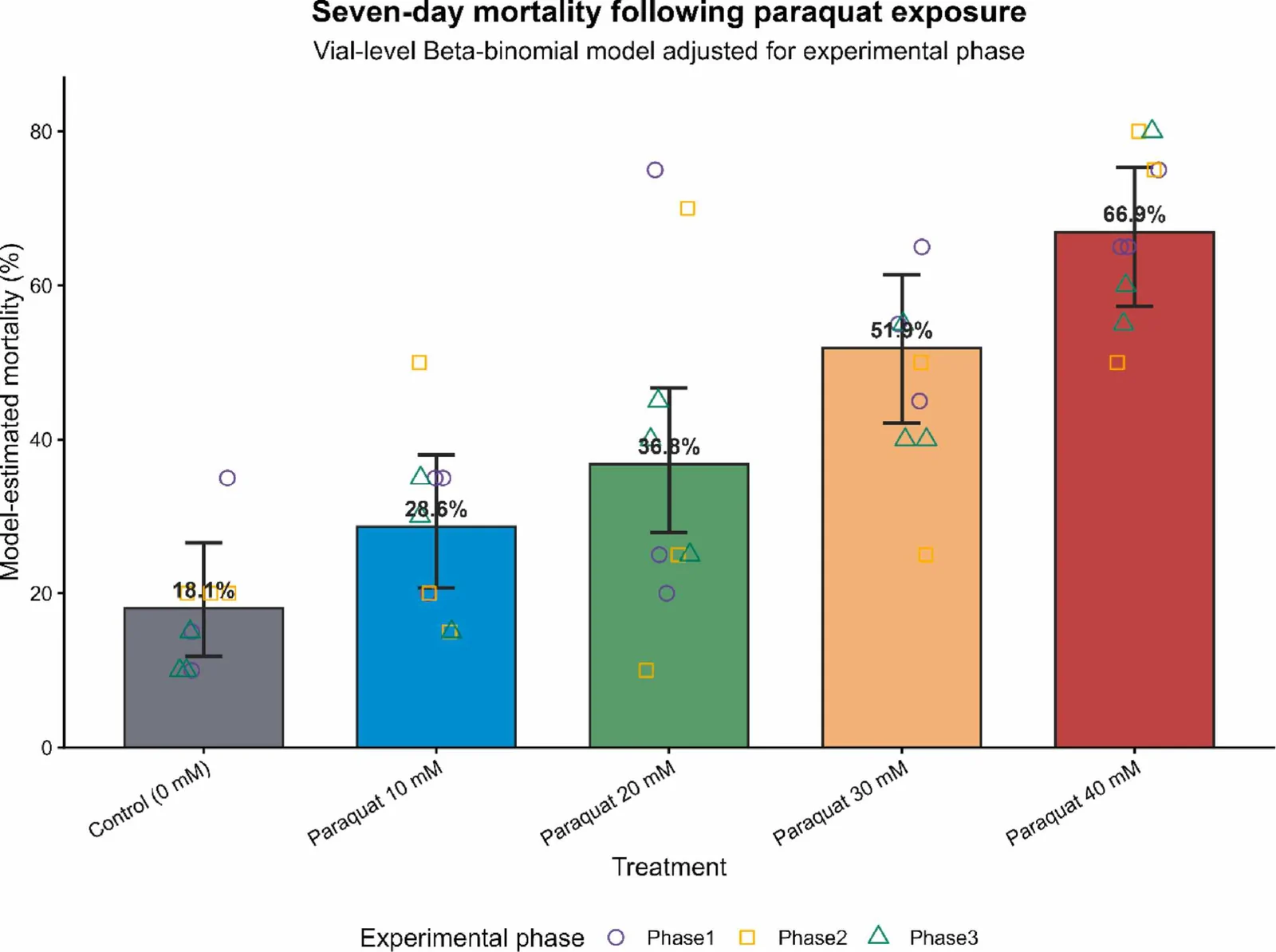
Phase-adjusted seven-day mortality of adult *Drosophila melanogaster* following paraquat exposure. *Bars represent mortality probabilities estimated using a vial-level beta-binomial model adjusted for experimental phase; error bars indicate 95% confidence intervals. Overlaid symbols represent mortality in individual replicate vials and distinguish the three independent experimental phases. Each treatment comprised nine replicate vials containing 20 flies each.*

#### 3.1.2 Concentration–mortality relationship and LC₅₀

When paraquat concentration was modelled as a continuous variable, mortality increased significantly with increasing concentration. Each 10 mM increase in paraquat was associated with a 1.72-fold increase in the odds of seven-day mortality (95% CI: 1.50–1.98; *p* < 0.001; Table 2). The phase-adjusted model estimated an LC₅₀ of 28.1 mM (95% bootstrap CI: 24.7–32.6 mM; Fig. 2). The LC₅₀ occurred within the experimentally tested concentration range and was supported by 5000 bootstrap iterations.

**Table 2:** Phase-adjusted paraquat concentration–mortality trend and LC₅₀ **estimate**.

Parameter	Estimate	95% CI	*p*-value
Odds ratio per 10 mM increase	1.72	1.50–1.98	< 0.001
Model-based LC₅₀	28.1 mM	24.7–32.6 mM	—

**Note:** The concentration trend was estimated using a phase-adjusted beta-binomial model. The LC₅₀ and its 95% CI were derived from model predictions using 5000 parametric bootstrap simulations.

**Fig. 2:**
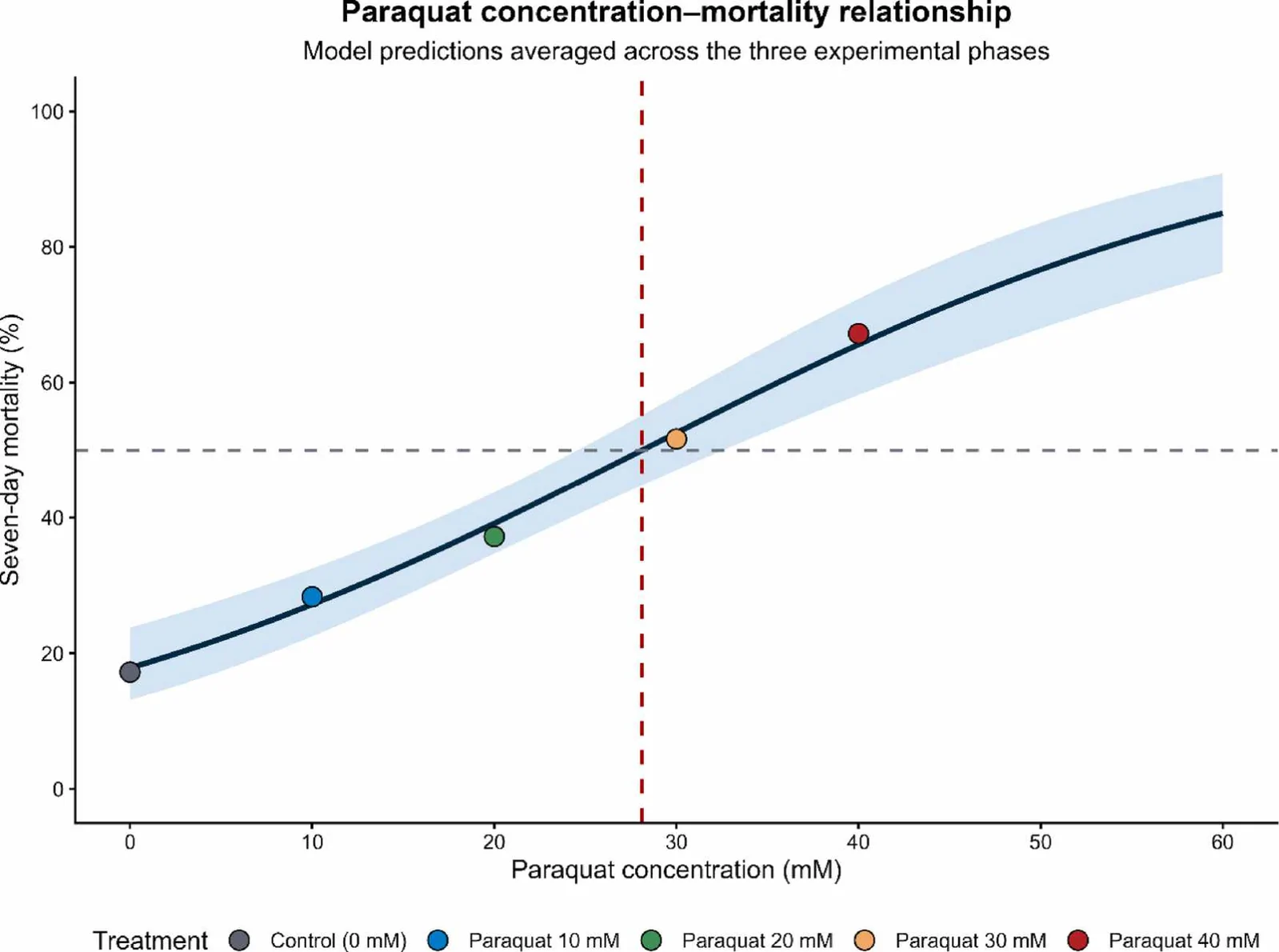
Phase-adjusted concentration–mortality relationship for paraquat-exposed adult *Drosophila melanogaster*. *Points represent observed seven-day mortality at the tested paraquat concentrations. The solid curve represents phase-adjusted model predictions, and the shaded region indicates the 95% confidence interval. The horizontal dashed line represents 50% mortality, and the vertical dashed line indicates the estimated LC₅₀ of 28.1 mM. Predictions beyond the tested range of 0–40 mM should be regarded as extrapolations.*

#### 3.1.3 Temporal progression of paraquat-associated mortality

Cumulative mortality increased throughout the seven-day exposure period and accumulated more rapidly at higher paraquat concentrations (Fig. 3). By Day 3, cumulative mortality was 5.6% in control and 13.9%, 17.8%, 25.6%, and 31.1% at 10, 20, 30, and 40 mM, respectively. By Day 7, the corresponding mortality values were 17.2%, 28.3%, 37.2%, 51.7%, and 67.2%. The discrete-time hazard model showed progressively increasing mortality hazards with paraquat concentration (Table 3). Relative to the control, mortality hazard was increased at 10 mM (HR = 1.81, 95% CI: 1.04–3.15; *p* = 0.037), 20 mM (HR = 2.58, 95% CI: 1.50–4.42; *p* < 0.001), 30 mM (HR = 4.09, 95% CI: 2.42–6.90; *p* < 0.001), and 40 mM (HR = 5.78, 95% CI: 3.45–9.67; *p* < 0.001). Mortality hazards were higher on Days 6 and 7 than on Day 1. The treatment-by-day interaction was not significant (χ² = 22.76, df = 24, *p* = 0.534), indicating no evidence that the relative treatment effect changed substantially across exposure days.

**Fig. 3:**
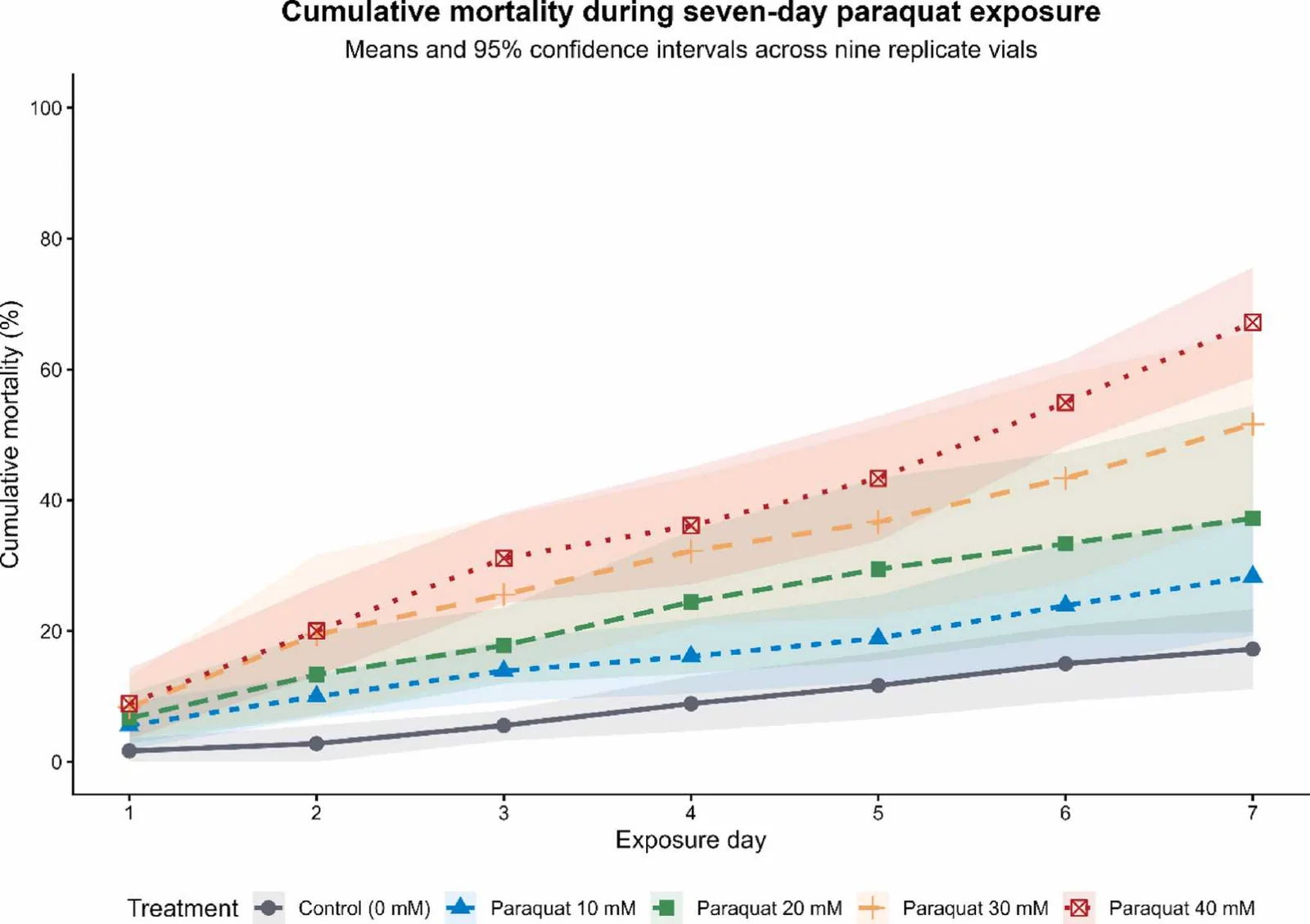
Cumulative mortality of adult *Drosophila melanogaster* during seven-day paraquat exposure. *Lines represent mean cumulative mortality across nine replicate vials per treatment; shaded regions indicate 95% confidence intervals. Mortality accumulated progressively with exposure time and reached higher final levels with increasing paraquat concentration.*

**Table 3:** Discrete-time mortality hazard during seven-day paraquat exposure.

Model term	Hazard ratio	95% CI	*p*-value
Treatment effect			
10 mM vs control	1.81	1.04–3.15	0.037
20 mM vs control	2.58	1.50–4.42	< 0.001
30 mM vs control	4.09	2.42–6.90	< 0.001
40 mM vs control	5.78	3.45–9.67	< 0.001
Exposure-day effect			
Day 2 vs Day 1	1.23	0.85–1.76	0.267
Day 3 vs Day 1	1.12	0.77–1.64	0.545
Day 4 vs Day 1	1.04	0.70–1.54	0.863
Day 5 vs Day 1	1.05	0.70–1.58	0.805
Day 6 vs Day 1	1.62	1.12–2.36	0.011
Day 7 vs Day 1	1.92	1.32–2.79	< 0.001

**Note:** Hazard ratios were estimated using a complementary log–log discrete-time hazard model accounting for repeated observations within replicate vials.

#### 3.1.4 Survival probability and mortality hazard

Kaplan–Meier analysis showed progressively lower survival with increasing paraquat concentration (Fig. 4). Survival distributions differed significantly among treatments (global log-rank p < 0.001). The phase-stratified Cox model included 900 flies and 363 mortality events and showed progressively increasing mortality hazards with concentration (Table 4). Compared with the control, mortality hazard was significantly elevated at 10 mM (HR = 1.78, 95% CI: 1.18–2.69; p = 0.006), 20 mM (HR = 2.53, 95% CI: 1.46–4.40; p < 0.001), 30 mM (HR = 3.90, 95% CI: 2.50–6.06; p < 0.001), and 40 mM (HR = 5.53, 95% CI: 3.98–7.68; p < 0.001). The overall Cox treatment effect was significant (robust score test = 21.69, p = 0.0002), and the proportional-hazards assumption was satisfied (global test p = 0.100). Specifically, 10 mM was not significantly different from the control in the Dunnett-adjusted seven-day endpoint analysis (p = 0.220) but was significant in the Cox time-to-event analysis (p = 0.006). Thus, its classification as the operational seven-day endpoint NOEC applies specifically to the predefined seven-day endpoint mortality analysis and should not be interpreted as an absence of any detectable survival effect.

**Fig. 4:**
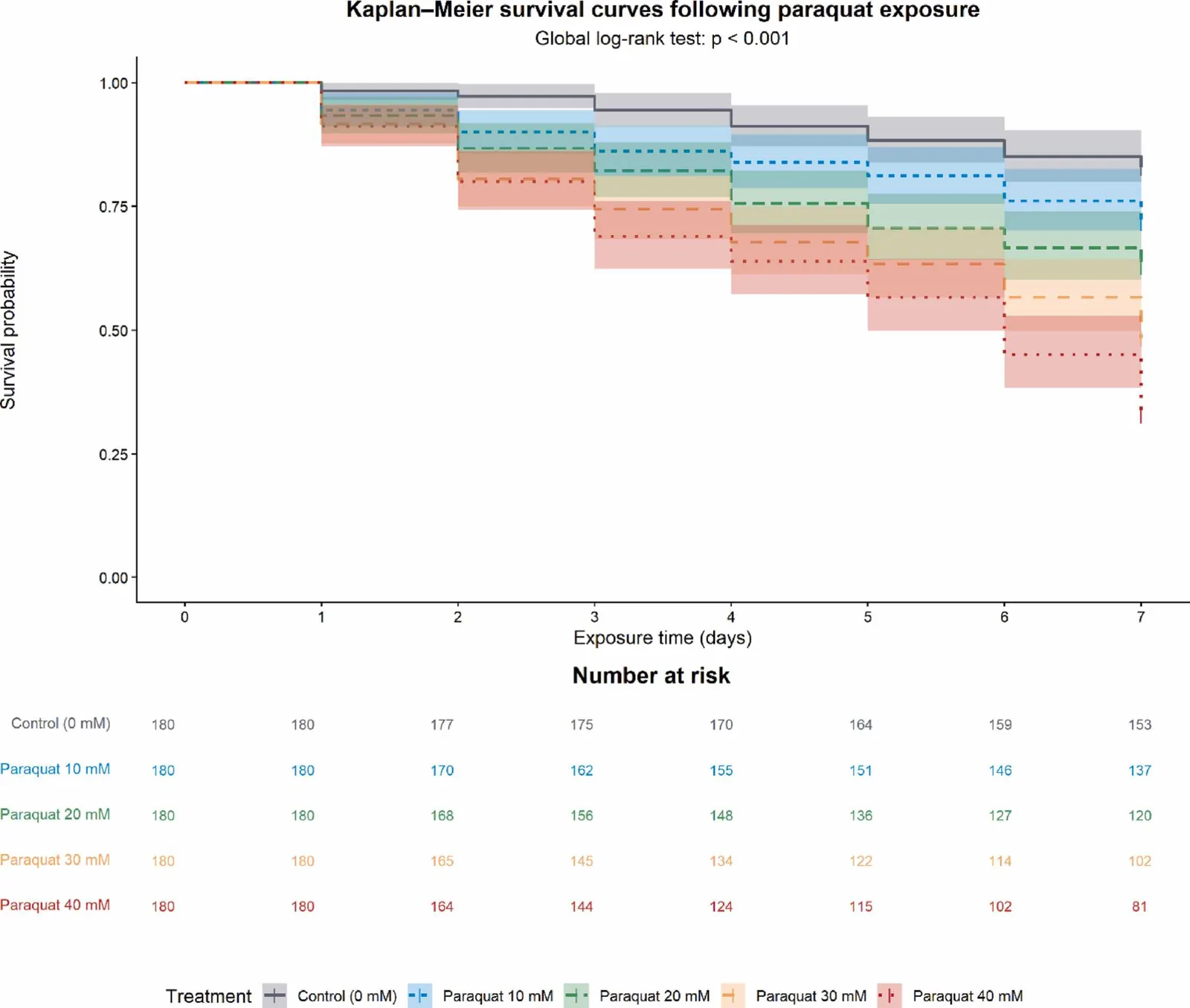
Kaplan–Meier survival curves of adult *Drosophila melanogaster* during seven-day paraquat exposure. Curves represent survival probabilities for the control and four paraquat concentrations, with shaded regions indicating 95% confidence intervals. The number-at-risk table shows flies remaining under observation at each time point. Survival decreased progressively with increasing paraquat concentration (global log-rank p < 0.001). Each treatment initially comprised 180 flies distributed among nine replicate vials.

**Table 4:** Survival analysis of adult *Drosophila melanogaster* during seven-day paraqu**at exposure**.

Analysis	Comparison/test	Estimate	95% CI	*p*-value
Cox model	10 mM vs control	1.78	1.18–2.69	0.006
Cox model	20 mM vs control	2.53	1.46–4.40	< 0.001
Cox model	30 mM vs control	3.90	2.50–6.06	< 0.001
Cox model	40 mM vs control	5.53	3.98–7.68	< 0.001
Global Cox test	Robust score test	21.69	—	0.0002
Proportional-hazards test	Global test	—	—	0.100

#### 3.1.5 Reproducibility across experimental phases and model adequacy

The progressive paraquat mortality pattern was observed across all three independent experimental phases (Fig. 5, Fig. 6). The treatment-by-phase interaction was not significant (χ² = 1.20, df = 8, *p* = 0.997), providing no evidence that the relative treatment effect differed among experimental runs. Model diagnostics showed no evidence of residual overdispersion (*p* = 0.632), non-uniform residuals (*p* = 0.636), or excess outliers (*p* = 1.000) for the endpoint model. Similarly, the discrete-time hazard model showed no evidence of residual dispersion (*p* = 0.532) or non-uniformity (*p* = 0.121).

**Fig. 5:**
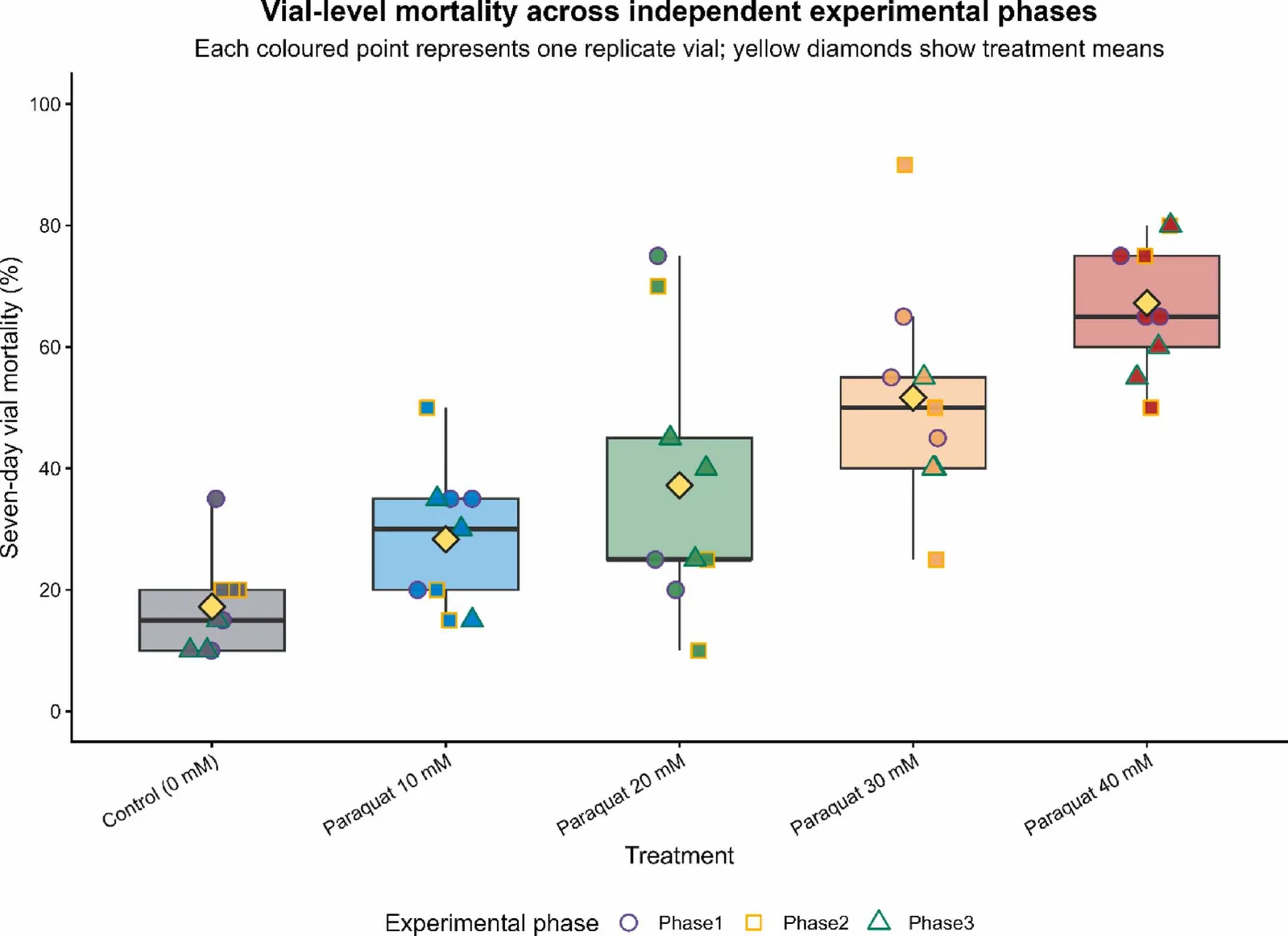
Vial-level seven-day mortality of adult *Drosophila melanogaster* across paraquat treatments and experimental phases. *Boxplots summarize mortality among nine replicate vials per treatment. Symbols represent individual replicate vials and identify experimental phase; diamonds indicate descriptive treatment means.*

**Fig. 6:**
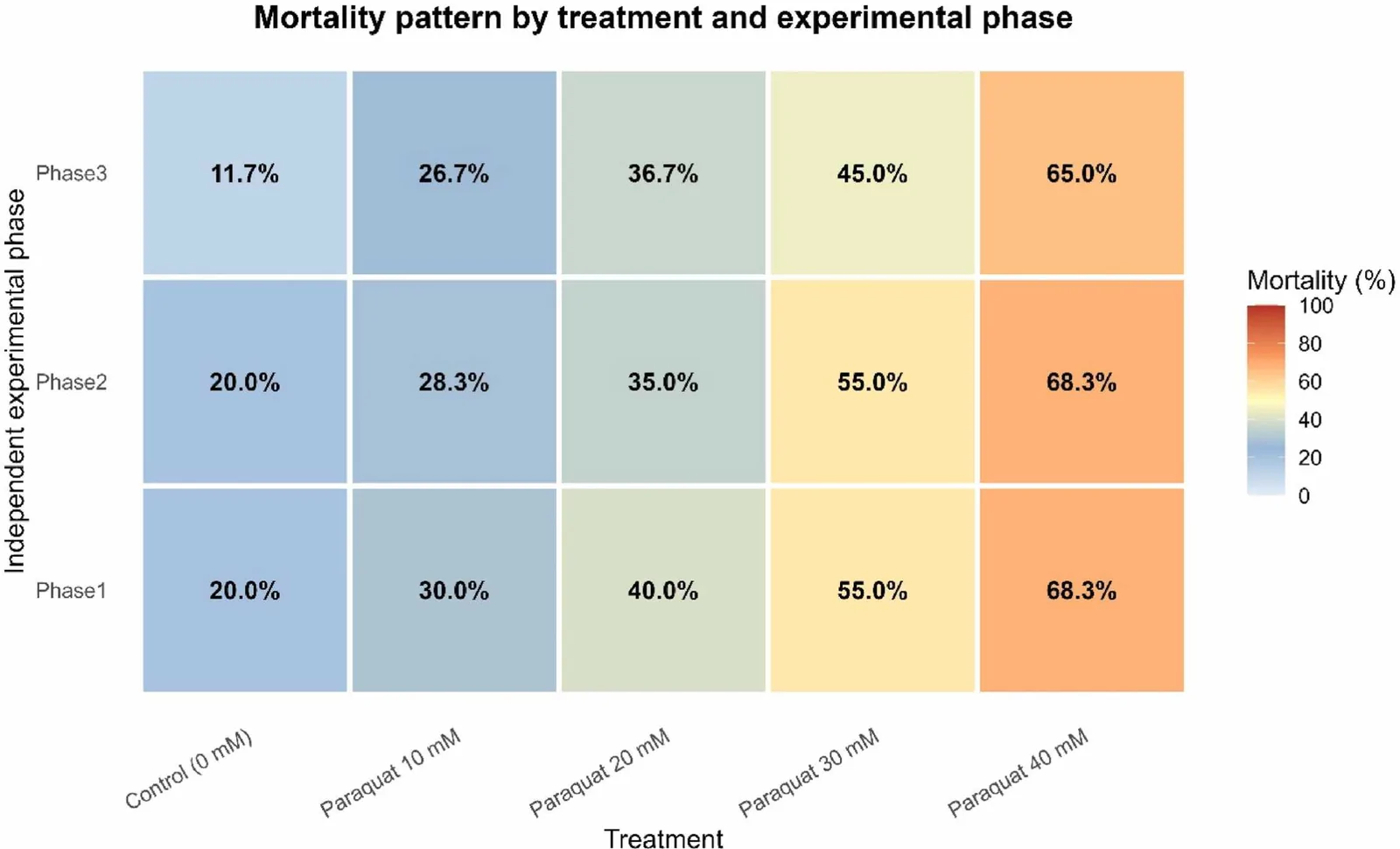
Phase-specific seven-day mortality following paraquat exposure. *Heatmap cells represent mortality percentages for each treatment and experimental phase, based on three replicate vials and 60 flies per phase–treatment combination. The progressive increase in mortality with paraquat concentration was evident across all three phases.*

### 3.2 Glyphosate-based formulation toxicity

#### 3.2.1 Seven-day endpoint mortality

The Roundup 360 SL experiment comprised 36 replicate vials and 720 flies, with nine vials and 180 flies per treatment across three independent phases. Seven-day mortality was 13.3% in control and 22.2%, 27.8%, and 26.7% at 0.5, 1.0, and 2.0 g/L GAE, respectively (Table 5; Fig. 7); the corresponding deaths were 24, 40, 50, and 48 of 180 flies. The phase-adjusted binomial model estimated mortality of 12.9%, 21.8%, 27.3%, and 26.2%, respectively. Dunnett-adjusted endpoint mortality was not significantly increased at 0.5 g/L GAE (OR = 1.87, 95% CI 0.96–3.67; p = 0.075) but was significantly increased at 1.0 g/L GAE (OR = 2.53, 95% CI 1.32–4.87; p = 0.002) and 2.0 g/L GAE (OR = 2.39, 95% CI 1.24–4.61; p = 0.005). Accordingly, the operational seven-day endpoint NOEC was 0.5 g/L GAE and the LOEC was 1.0 g/L GAE. Observed mortality differed by only 1.1 %age points between 1.0 and 2.0 g/L GAE. Thus, the two highest Roundup treatments produced similar endpoint mortality rather than a progressive increase across the entire tested concentration series.

**Table 5:** Seven-day mortality of adult *Drosophila melanogaster* following exposure to a glyphosate-based formu**lation**.

Roundup 360 SL concentration (g/L GAE)	Replicate vials (n)	Deaths/initial flies	Observed mortality (%)	Model-estimated mortality, % (95% CI)	Dunnett-adjusted OR (95% CI)	Adjusted *p*-value
Control (0)	9	24/180	13.3	12.9 (8.8–18.7)	Reference	—
0.5	9	40/180	22.2	21.8 (16.3–28.4)	1.87 (0.96–3.67)	0.075
1.0	9	50/180	27.8	27.3 (21.3–34.4)	2.53 (1.32–4.87)	0.002
2.0	9	48/180	26.7	26.2 (20.3–33.2)	2.39 (1.24–4.61)	0.005

Note: OR, odds ratio; CI, confidence interval; GAE, glyphosate acid equivalent. Estimates were obtained from a phase-adjusted binomial generalized linear model. Adjusted p-values are Dunnett comparisons with the concurrent control. Roundup concentrations are target g/L GAE reconstructed from the recorded preparation volumes.

**Fig. 7:**
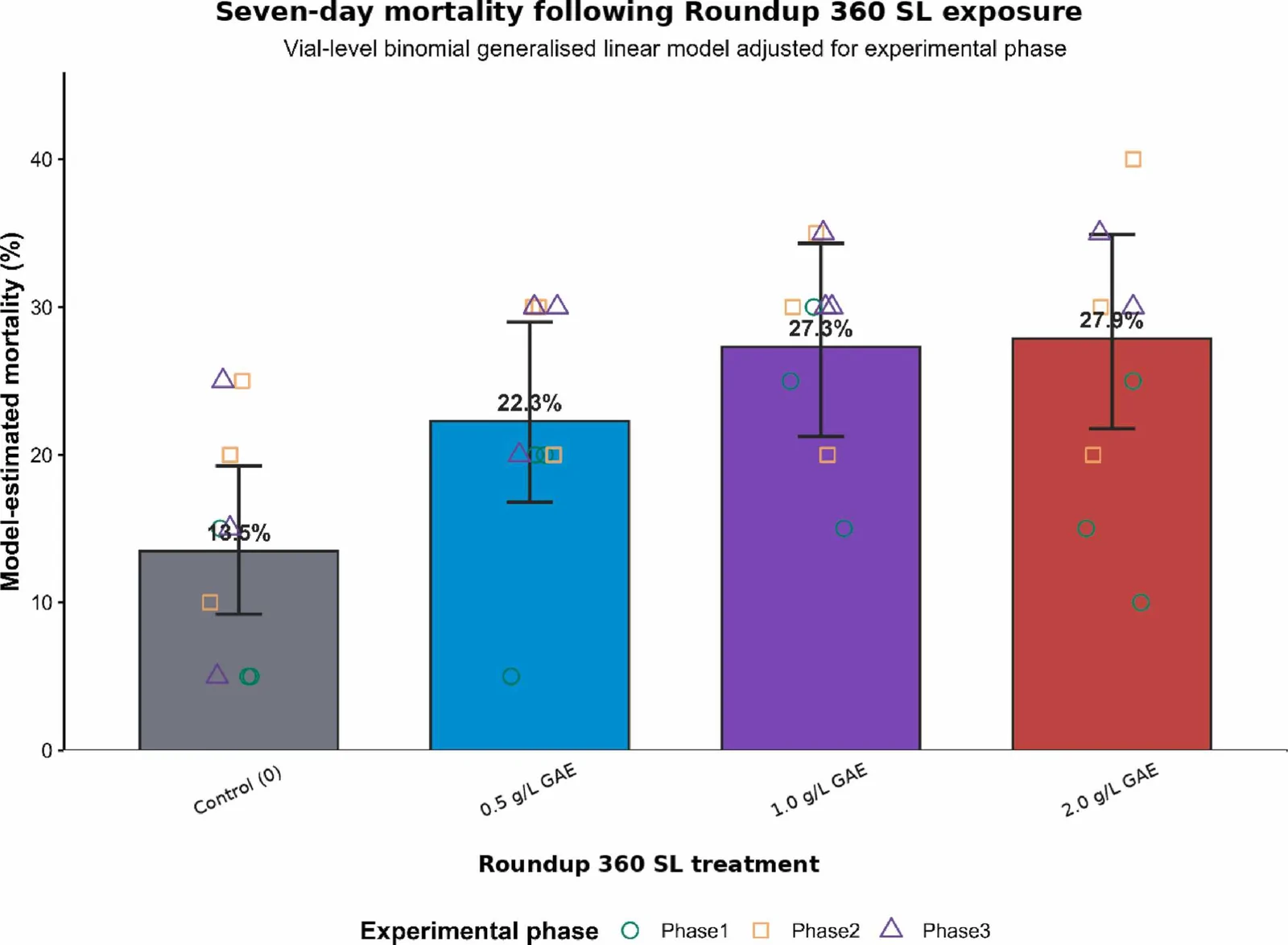
Phase-adjusted seven-day mortality of adult *Drosophila melanogaster* following exposure to Roundup 360 SL. *Bars represent mortality probabilities estimated using a vial-level binomial generalized linear model adjusted for experimental phase; error bars indicate 95% confidence intervals. Symbols show replicate-vial mortality and distinguish the three experimental phases. Each treatment comprised nine replicate vials of 20 flies. Concentrations are target g/L glyphosate acid equivalent (GAE).*

#### 3.2.2 Exploratory concentration–mortality pattern

Treatment was retained as a categorical factor in the primary endpoint analysis because only three non-zero GAE concentrations were tested. Linear and quadratic concentration models were fitted only as exploratory assessments of response shape (Table 6; Fig. 8). The phase-adjusted linear model indicated an overall increase in mortality with increasing GAE concentration; each 1 g/L increase was associated with a 1.42-fold increase in the odds of seven-day mortality (95% CI 1.12–1.80; p = 0.003). The quadratic model fitted the observed pattern better than the linear model (likelihood-ratio p = 0.016; AIC 138.24 vs 142.07), with a negative squared-concentration coefficient (β = −0.503, 95% CI −0.913 to −0.092; p = 0.016). This curvature reflects flattening between 1.0 and 2.0 g/L GAE, but the limited number of non-zero concentrations does not establish a reproducible non-monotonic response. No beneficial or stimulatory low-concentration response was observed; therefore, the findings do not demonstrate hormesis. An LC50 was not estimated because the maximum observed mortality was 27.8%, making an estimate dependent on extrapolation beyond the tested range.

**Table 6:** Exploratory concentration–mortality models for seven-day mortality following exposure to a glyphosate-based formulation.

Analysis	Parameter or comparison	Estimate	95% CI	*p*-value
Linear model	GAE concentration coefficient (per 1 g/L)	0.352	0.117–0.587	0.003
Linear model	OR per 1 g/L GAE increase	1.42	1.12–1.80	0.003
Quadratic model	Linear GAE concentration coefficient	1.433	0.509–2.358	0.002
Quadratic model	Squared GAE concentration coefficient	−0.503	−0.913 to −0.092	0.016
Model comparison	Linear vs quadratic	—	—	0.016
Model fit	Linear-model AIC	142.07	—	—
Model fit	Quadratic-model AIC	138.24	—	—
LC₅₀ assessment	Maximum observed mortality	27.8%	—	Not estimated

Note: OR, odds ratio; CI, confidence interval; AIC, Akaike information criterion; GAE, glyphosate acid equivalent. Linear and quadratic models were exploratory; the categorical treatment model was the primary inferential analysis. A better-fitting quadratic model indicates curvature in the fitted relationship but does not establish hormesis or a reproducible non-monotonic biological response.

**Fig. 8:**
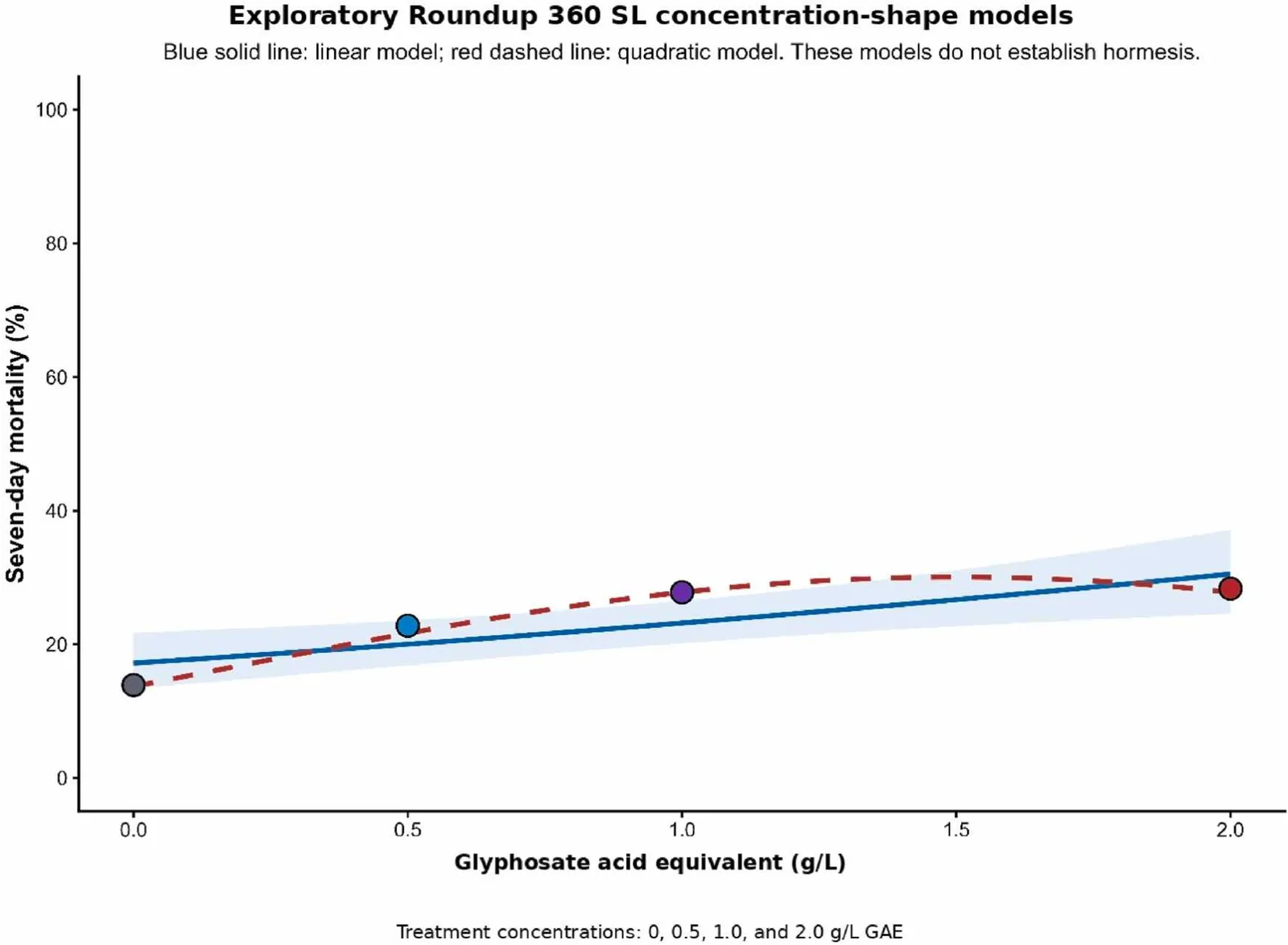
Exploratory linear and quadratic concentration–mortality relationships for adult *Drosophila melanogaster* following seven-day exposure to Roundup 360 SL. *Concentrations are target g/L glyphosate acid equivalent (GAE). The quadratic model fitted the observed pattern better than the linear model (AIC 138.24 vs 142.07; likelihood-ratio p = 0.016), reflecting upper-range flattening. This exploratory curvature is not interpreted as evidence of hormesis or a confirmed non-monotonic response.*

#### 3.2.3 Temporal progression of mortality

Cumulative mortality increased throughout the seven-day exposure period in all groups, although temporal patterns differed among treatments (Fig. 9, Fig. 10). By Day 3, cumulative mortality was 5.6% in control and 10.6%, 8.3%, and 6.1% at 0.5, 1.0, and 2.0 g/L GAE, respectively. By Day 5, mortality had reached 6.1%, 17.8%, 18.3%, and 18.9%, respectively, and by Day 7 it reached 13.3%, 22.2%, 27.8%, and 26.7%. In the additive discrete-time hazard model, average mortality hazard across the seven-day period was significantly elevated at 0.5 g/L GAE (HR = 1.81, 95% CI 1.09–3.00; p = 0.022), 1.0 g/L GAE (HR = 2.25, 95% CI 1.38–3.66; p = 0.001), and 2.0 g/L GAE (HR = 2.18, 95% CI 1.33–3.56; p = 0.002) relative to control (Table 7). Because treatment effects varied over time, these hazard ratios represent average effects across the full observation period. Mortality hazard was also higher on Days 5 and 6 than on Day 1.

**Fig. 9:**
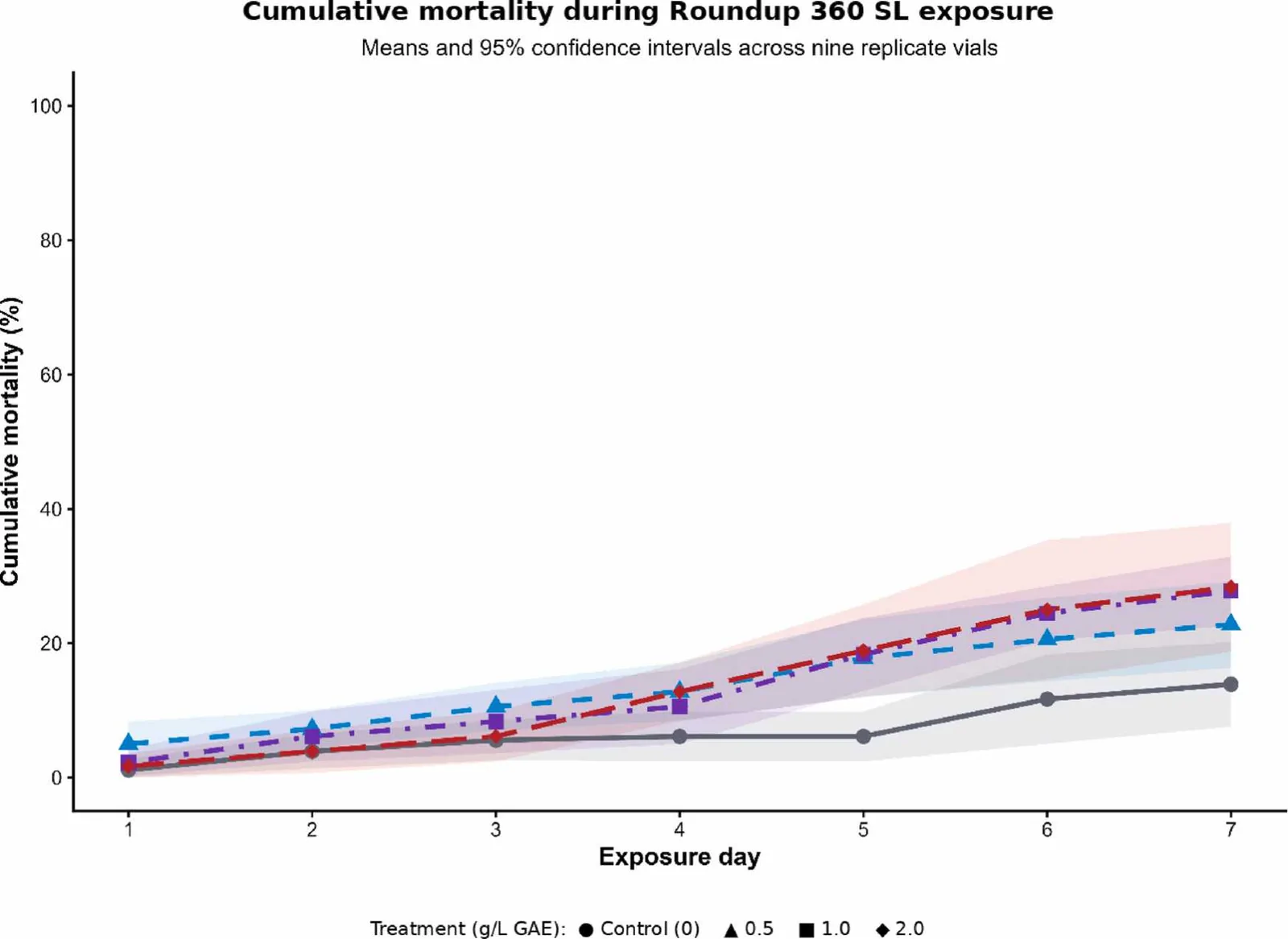
Cumulative mortality of adult *Drosophila melanogaster* during seven-day exposure to Roundup 360 SL. *Lines represent mean cumulative mortality across nine replicate vials per treatment; shaded regions indicate 95% confidence intervals. Concentrations are target g/L glyphosate acid equivalent (GAE).*

**Fig. 10:**
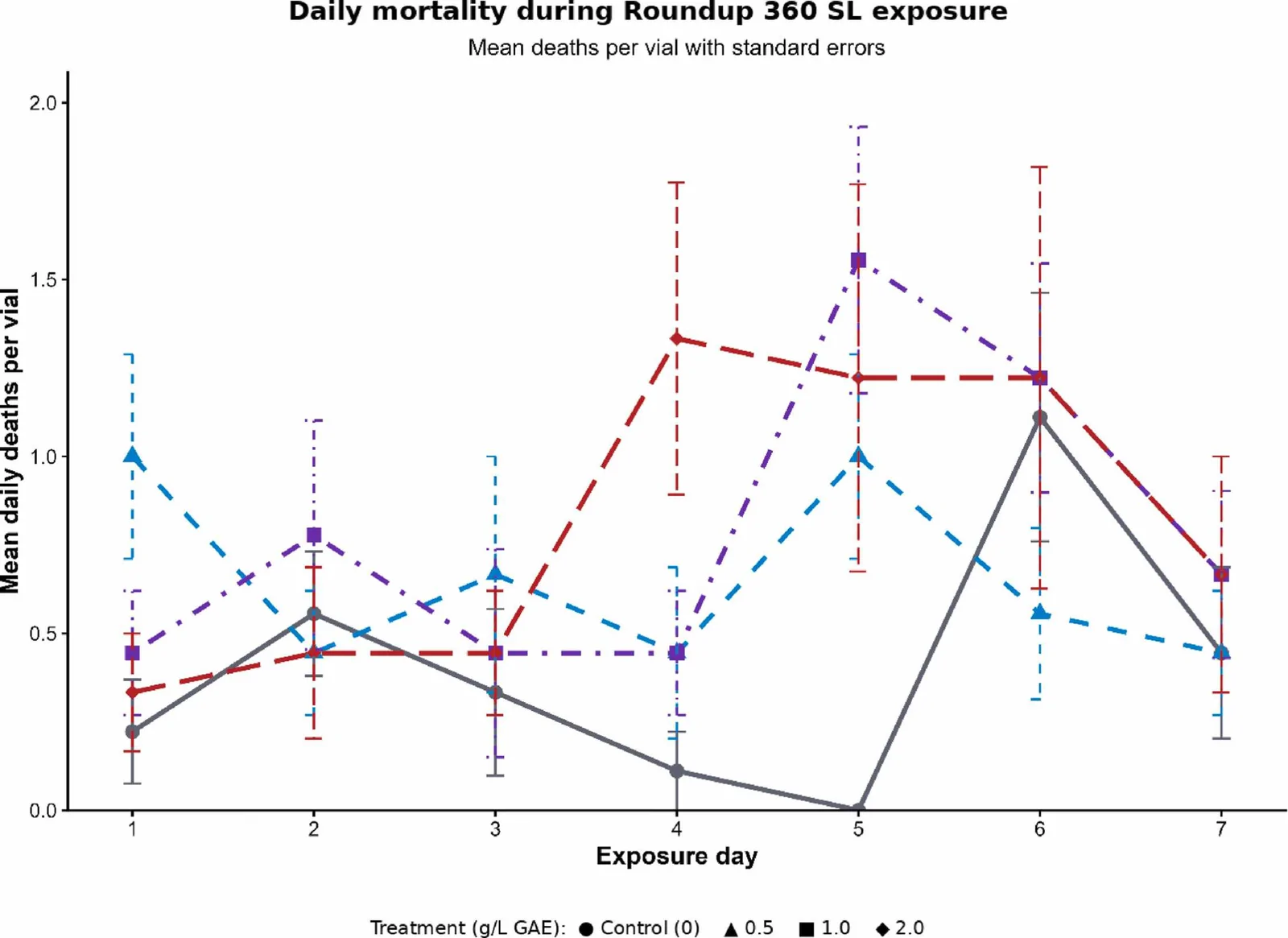
Daily mortality of adult *Drosophila melanogaster* during seven-day exposure to Roundup 360 SL. *Points and lines represent mean daily deaths per replicate vial; error bars indicate standard errors. Concentrations are target g/L glyphosate acid equivalent (GAE).*

**Table 7:** Discrete-time mortality hazard during seven-day exposure to a glyphosate-based formula**tion**.

Model term	Hazard ratio	95% CI	*p*-value
Treatment effect			
0.5 g/L GAE vs control	1.81	1.09–3.00	0.022
1.0 g/L GAE vs control	2.25	1.38–3.66	0.001
2.0 g/L GAE vs control	2.18	1.33–3.56	0.002
Exposure-day effect			
Day 2 vs Day 1	1.14	0.61–2.16	0.679
Day 3 vs Day 1	1.00	0.52–1.94	1.000
Day 4 vs Day 1	1.28	0.68–2.39	0.449
Day 5 vs Day 1	2.18	1.23–3.86	0.008
Day 6 vs Day 1	2.19	1.23–3.90	0.008
Day 7 vs Day 1	1.44	0.76–2.72	0.263
Experimental-phase effect			
Phase 2 vs Phase 1	1.58	1.05–2.39	0.028
Phase 3 vs Phase 1	1.89	1.27–2.81	0.002

#### 3.2.4 Survival probability and mortality hazard

The survival analysis included 720 flies and 162 mortality events. Seven-day survival was 86.7% in the control and 77.8%, 72.2%, and 73.3% at 0.5, 1.0, and 2.0 g/L GAE, respectively. Kaplan–Meier curves showed lower survival in formulation-exposed groups than in controls (Fig. 11), and the global log-rank test indicated different survival distributions (p = 0.005). In the phase-stratified Cox model with vial-clustered robust standard errors, mortality hazard was increased at 0.5 g/L GAE (HR = 1.80, 95% CI 1.21–2.68; p = 0.004), 1.0 g/L GAE (HR = 2.24, 95% CI 1.55–3.25; p < 0.001), and 2.0 g/L GAE (HR = 2.18, 95% CI 1.47–3.22; p < 0.001) (Table 8). The similar hazard ratios at 1.0 and 2.0 g/L GAE were consistent with their similar endpoint mortality. The proportional-hazards assumption was supported (global p = 0.168).

**Fig. 11:**
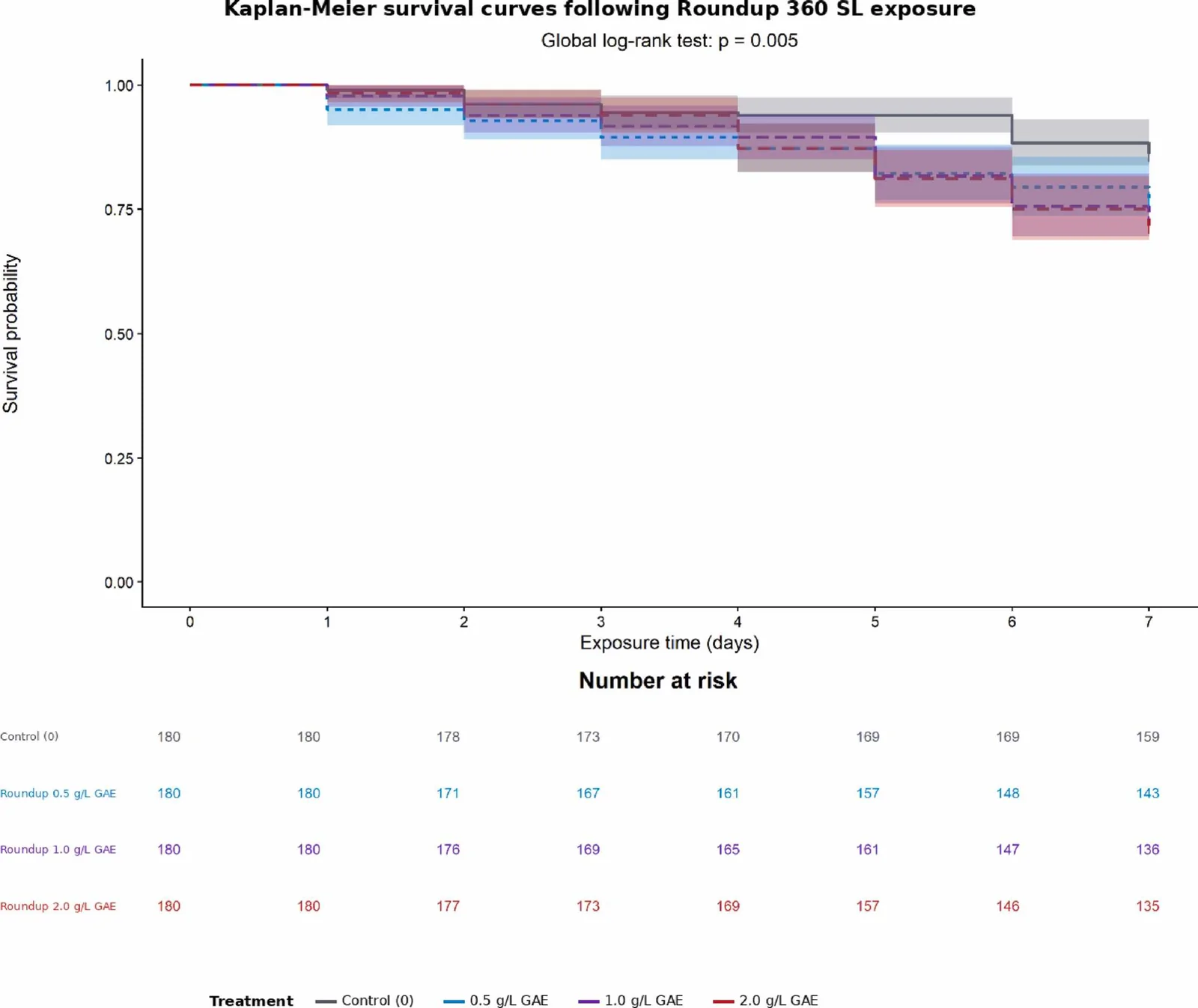
Kaplan–Meier survival curves of adult *Drosophila melanogaster* during seven-day exposure to Roundup 360 SL. Curves represent survival probabilities for the control and three treatment concentrations; shaded regions indicate 95% confidence intervals. The number-at-risk table presents flies remaining under observation. Survival distributions differed among treatments (global log-rank p = 0.005). Concentrations are target g/L glyphosate acid equivalent (GAE).

**Table 8:** Survival analysis of adult *Drosophila melanogaster* during seven-day exposure to a glyphosate-based formulation.

Analysis	Comparison/test	Estimate	95% CI	*p*-value
Cox model	0.5 g/L GAE vs control	1.80	1.21–2.68	0.004
Cox model	1.0 g/L GAE vs control	2.24	1.55–3.25	< 0.001
Cox model	2.0 g/L GAE vs control	2.18	1.47–3.22	< 0.001
Global Cox test	Wald test	—	—	< 0.001
Global log-rank test	Descriptive comparison	—	—	0.005
Proportional-hazards test	Global test	—	—	0.168

#### 3.2.5 Reproducibility across experimental phases and model adequacy

Mortality varied among the three experimental phases (Fig. 12, Fig. 13). In Phase 1, seven-day mortality was 8.3% in controls and 15.0%, 23.3%, and 16.7% at 0.5, 1.0, and 2.0 g/L GAE, respectively. Corresponding values were 16.7%, 25.0%, 28.3%, and 25.0% in Phase 2 and 15.0%, 26.7%, 31.7%, and 38.3% in Phase 3. Although overall mortality differed among phases, the treatment-by-phase interaction was not significant, providing no statistical evidence that the relative treatment pattern differed among runs. Simulation-based diagnostics showed no important residual dispersion, non-uniformity, or excess outliers for the endpoint or discrete-time hazard models; the proportional-hazards assumption was also supported for the Cox model.

**Fig. 12:**
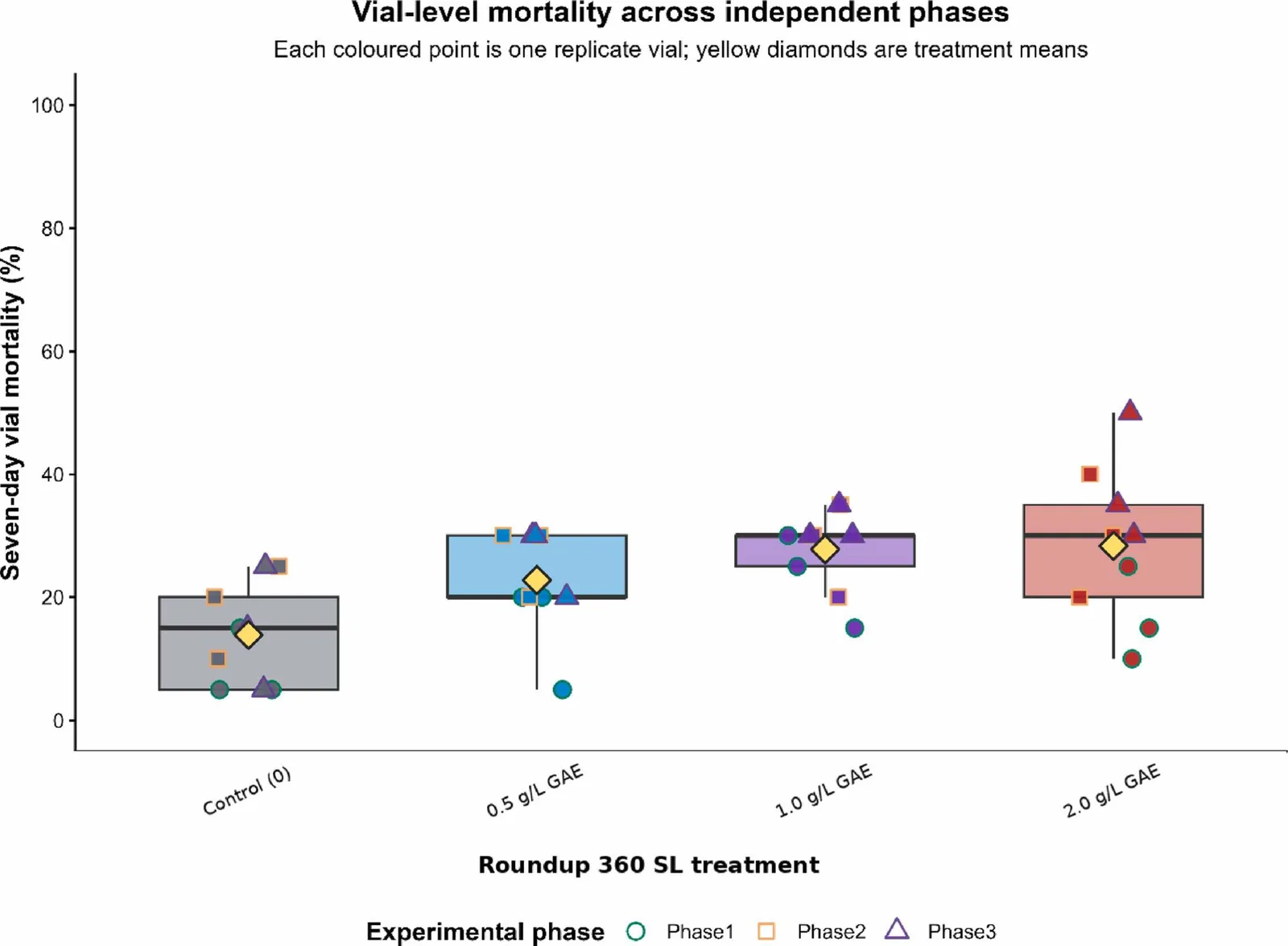
Vial-level seven-day mortality of adult *Drosophila melanogaster* following exposure to Roundup 360 SL across three independent experimental phases. Boxplots summarize mortality among nine replicate vials per treatment; individual symbols identify replicate vials and phase, and diamonds indicate descriptive treatment means. Concentrations are target g/L glyphosate acid equivalent (GAE).

**Fig. 13:**
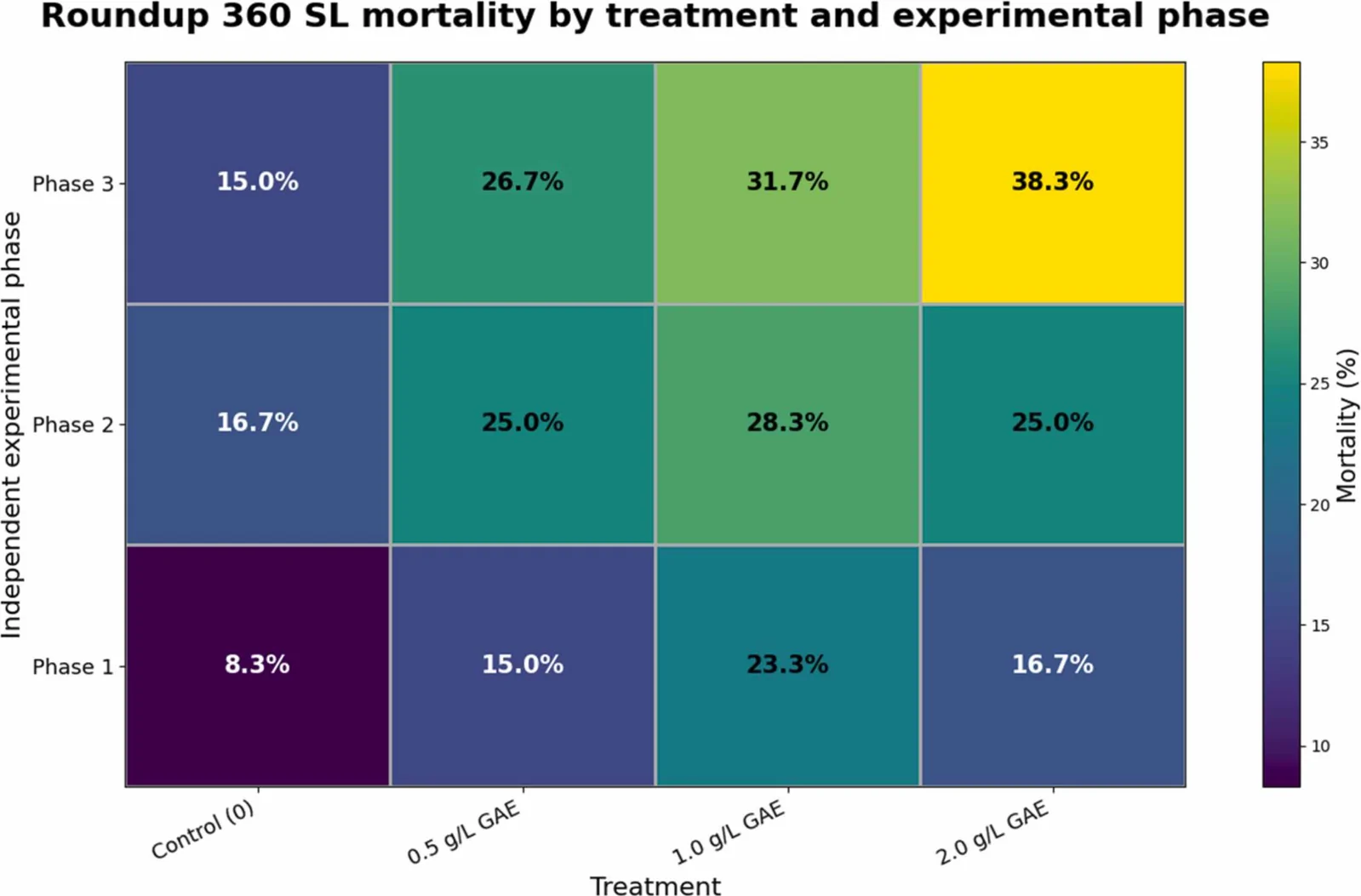
Phase-specific seven-day mortality of adult *Drosophila melanogaster* following exposure to Roundup 360 SL. Heatmap cells represent mortality percentages for each treatment and experimental phase, based on three replicate vials and 60 flies per phase–treatment combination. Concentrations are target g/L glyphosate acid equivalent (GAE).

### 3.3 Comparative mortality and survival patterns of paraquat and the glyphosate-based formulation

Both experiments showed treatment-associated increases in mortality relative to their concurrent controls, but the within-experiment concentration–mortality patterns differed (Table 9). Paraquat mortality increased progressively from 17.2% in the control to 67.2% at 40 mM; the operational seven-day endpoint NOEC and LOEC were 10 and 20 mM, respectively, and the LC50 was 28.1 mM (95% bootstrap CI 24.7–32.6). For Roundup 360 SL, mortality increased from 13.3% in the control to 22.2% at 0.5 g/L GAE and 27.8% at 1.0 g/L GAE, then remained similar at 26.7% at 2.0 g/L GAE. The corresponding operational seven-day endpoint NOEC and LOEC were 0.5 and 1.0 g/L GAE. An LC50 was not estimated for Roundup because mortality remained below 50% throughout the tested range.

**Table 9:** Summary of mortality and survival outcomes for paraquat and a glyphosate-based formulation in adult *Drosophila melanogaster*.

Outcome	Paraquat	Glyphosate-based formulation
Tested treatments	0, 10, 20, 30, 40 mM	0, 0.5, 1.0, 2.0 g/L GAE
Replicate vials per treatment	9	9
Flies per treatment	180	180
Control mortality	17.2%	13.3%
Highest observed mortality	67.2% at 40 mM	27.8% at 1.0 g/L GAE
Treatments significantly different from control in endpoint analysis	20, 30, and 40 mM	1.0 and 2.0 g/L GAE
Operational endpoint NOEC	10 mM	0.5 g/L GAE
Operational endpoint LOEC	20 mM	1.0 g/L GAE
LC₅₀	28.1 mM (95% CI: 24.7–32.6)	Not estimated
Cox HR at lowest non-zero treatment	1.78	1.80
Cox HR at highest tested treatment	5.53	2.18
Overall mortality pattern	Progressive increase with concentration	Increase to 1.0 g/L GAE, then similar mortality at 2.0 g/L GAE
Direct potency comparison	Not appropriate	Not appropriate

Note: NOEC and LOEC refer specifically to Dunnett-adjusted seven-day endpoint mortality comparisons with the respective concurrent control. HR, hazard ratio; CI, confidence interval; GAE, glyphosate acid equivalent. Direct relative-potency comparison was not performed because the two products were expressed on different concentration bases and differed in formulation composition.

Time-to-event analyses showed the same broad contrast in response shape. Paraquat Cox mortality hazards increased progressively across 10, 20, 30, and 40 mM (HR 1.78, 2.53, 3.90, and 5.53, respectively), whereas Roundup hazard ratios were 1.80, 2.24, and 2.18 at 0.5, 1.0, and 2.0 g/L GAE. These results describe patterns within each experiment and do not provide a valid relative-potency ranking. Paraquat was expressed in mM paraquat equivalent and Roundup in g/L glyphosate acid equivalent, the products differed in formulation composition, and individual dietary intake was not measured.

## 4 Discussion

### 4.1 Principal findings

This study identified distinct within-experiment mortality patterns for two commercial herbicide formulations in adult male Drosophila melanogaster. Paraquat produced a progressive increase in endpoint mortality and mortality hazard across 10–40 mM. Roundup 360 SL increased mortality relative to control, but the 1.0 and 2.0 g/L GAE groups had similar endpoint mortality and Cox hazards. The record establishes that the Roundup treatment labels represent g/L glyphosate acid equivalent. These findings support comparison of response patterns within each experiment, but not a numerical ranking of relative chemical potency.

### 4.2 Concentration-related toxicity of paraquat

Paraquat produced a clear progressive concentration–mortality relationship, with seven-day mortality increasing from 17.2% in controls to 67.2% at 40 mM and a model-based LC50 of 28.1 mM within the tested range. The progressive hazard increase was consistent with this endpoint pattern. Paraquat toxicity is biologically compatible with redox cycling and oxidative stress [2], [4], and studies have reported survival, locomotor, oxidative, and neurotoxic effects after paraquat exposure [6], [18]. These mechanisms were not measured and therefore should not be inferred as the direct cause of mortality. The operational seven-day endpoint NOEC of 10 mM is also endpoint-specific: 10 mM was not significant in the Dunnett-adjusted Day-7 analysis (p = 0.220) but was associated with increased mortality hazard in the Cox analysis (p = 0.006).

### 4.3 Toxicity of the glyphosate-based formulation

Roundup 360 SL increased seven-day mortality from 13.3% in controls to 27.8% at 1.0 g/L GAE, while mortality at 2.0 g/L GAE was similar (26.7%). The corresponding Cox hazard ratios were likewise similar at the two highest concentrations. The better fit of the exploratory quadratic model therefore describes upper-range flattening, not proof of an inverted-U response. Only three non-zero concentrations were tested, and no beneficial or stimulatory low-concentration effect was observed; consequently, the data do not demonstrate hormesis. Potential explanations for the plateau include altered feeding, avoidance or palatability of treated food, or concentration-dependent changes in formulation availability, but none of these mechanisms were measured in this study and they remain hypotheses for future testing.

### 4.4 Interpretation of Roundup formulation effects

The Roundup findings must be interpreted at the formulation level. The product contains glyphosate potassium salt plus unspecified co-formulants, and no pure-glyphosate or matched co-formulant control was included. Commercial glyphosate formulations can differ toxicologically from glyphosate alone because co-formulants may alter biological effects [19], [20], [21]. Drosophila studies likewise support formulation-specific effects, including adverse reproductive or survival outcomes after Roundup exposure [9], [12]. Accordingly, the present results cannot identify which component of Roundup 360 SL produced the observed mortality.

### 4.5 Strengths, limitations and future research

A major strength was the explicit replicate-vial design across three independent biological phases, with vial-level endpoint models that account for the shared exposure environment. Daily mortality and survival modelling provided complementary time-to-event information. Limitations include the small number of non-zero Roundup concentrations, use of commercial products without analytical verification of exposure media, unavailable detailed co-formulant and batch information, lack of pure-active-ingredient controls, absence of food-consumption and internal-dose measurements, and use of only adult male Oregon K flies. These limitations restrict mechanistic interpretation and generalization.

Future experiments should verify exposure concentrations analytically, include pure glyphosate and formulation/co-formulant controls, and test additional Roundup concentrations around and above 1–2 g/L GAE to determine whether the apparent plateau is reproducible. Measurements of food consumption, internal pesticide burden, oxidative stress, mitochondrial function, and locomotor performance would help distinguish toxicodynamic effects from altered exposure. The present data support a progressive concentration–mortality relationship for paraquat and an increase followed by upper-range flattening for Roundup 360 SL; they do not establish hormesis, glyphosate-specific toxicity, or a direct relative-potency ranking between the products.

## 5 Conclusions

Seven-day dietary exposure to TwigaQuat 200 SL produced a progressive paraquat concentration–mortality relationship in adult male *Drosophila melanogaster*, with an operational seven-day endpoint NOEC of 10 mM, LOEC of 20 mM, and LC50 of 28.1 mM under the present conditions. Roundup 360 SL also increased mortality and reduced survival at target concentrations expressed as 0.5–2.0 g/L glyphosate acid equivalent; mortality at 1.0 and 2.0 g/L GAE was similar, indicating upper-range flattening within the tested range rather than evidence of hormesis. Because a complete commercial formulation was tested without pure-glyphosate or co-formulant controls, the Roundup effects cannot be attributed specifically to glyphosate. The different exposure metrics, formulation compositions, and absence of measured intake preclude a direct relative-potency comparison. Additional chemically verified concentrations and exposure measurements are required to define the Roundup concentration–response relationship more completely.

## Author Contributions

Raphael Mwezi and Flora Stephano: Conceptualization, Methodology, Investigation, Formal analysis, Data curation, Writing - Original Draft, and Visualization. Maulilio Kipanyula and John-Mary Vianney: Conceptualization, Methodology, Investigation, Supervision, Writing - Review & Editing, and Validation. All authors contributed to interpretation of the findings, critically reviewed the manuscript, approved the final version, and agreed to be accountable for the work.

## CRediT authorship contribution statement

**Raphael Mwezi:** Writing – original draft, Visualization, Methodology, Investigation, Formal analysis, Data curation, Conceptualization. **Flora Stephano:** Writing – review & editing, Writing – original draft, Visualization, Supervision, Methodology, Investigation, Data curation, Conceptualization. **Maulilio Kipanyula:** Writing – review & editing, Validation, Methodology, Investigation, Conceptualization. **John-Mary Vianney:** Writing – review & editing, Visualization, Supervision, Methodology, Investigation, Conceptualization.

## Ethics Statement

This study involved Drosophila melanogaster and did not involve vertebrate animals or human participants. Experimental procedures were conducted in accordance with institutional laboratory guidelines and accepted principles of good scientific practice.

## Funding

This research received no external funding. The study was conducted as part of the corresponding author’s doctoral research at the Nelson Mandela African Institution of Science and Technology (10.13039/501100010483NM-AIST), Tanzania.

## Declaration of Competing Interest

The authors declare that they have no known competing financial interests or personal relationships that could have appeared to influence the work reported in this paper.

## Data Availability

Data will be made available on request.
